# Effects of atorvastatin on inflammatory markers, lipid profile, liver enzymes, and pulmonary function in patients with lung diseases: a systematic review and meta-analysis of randomized controlled trials

**DOI:** 10.1186/s40001-025-03782-y

**Published:** 2026-01-19

**Authors:** Samane Baseri, Morteza Izadi, Mina Alimohammadi, Seyedeh Mahdieh Khoshnazar, Reza Nikdel, Kiavash Hushmandi

**Affiliations:** 1 Student Research Committee, Baqiyatallah University of Medical Sciences, Tehran, Iran; 2 Health Research Center, Life Style Institute, Baqiyatallah University of Medical Sciences, Tehran, IR Iran; 3 Department of Immunology, School of Medicine, Shahid Beheshti University of Medical Sciences, Tehran, Iran; 4 Physiology Research Center, Institute of Neuropharmacology, Kerman University of Medical Sciences, Kerman, Iran; 5 Nephrology and Urology Research Center, Clinical Sciences Institute, Baqiyatallah University of Medical Sciences, Tehran, Iran

**Keywords:** Atorvastatin, Pulmonary disease, Inflammatory biomarkers, Lipid profile, Pulmonary function, Meta-analysis

## Abstract

**Background:**

Pulmonary diseases are important causes of morbidity globally. Atorvastatin's pleiotropic effects, which include anti-inflammatory and lipid-lowering properties, may be beneficial for individuals with respiratory diseases. This meta-analysis evaluated the atorvastatin's effect on inflammatory biomarkers, lipid profile, liver enzymes, and pulmonary function in lung disease patients.

**Methods:**

We systematically searched PubMed/MEDLINE, Scopus, Web of Science, Embase, CENTRAL, and Google Scholar for English-language RCTs until March 2025. The study evaluated inflammatory markers (CRP, IL-6, TNF-α), lipid profile (LDL, HDL, TC, TG), liver enzymes (ALT, AST), pulmonary function tests, and physical performance. Pooled weighted mean differences (WMDs) with 95% confidence intervals were calculated using random-effects models. Subgroup, heterogeneity, and publication bias analyses were conducted.

**Results:**

Seventeen RCTs (22 datasets; *n* = 1,344) on asthma, COPD, COVID-19, pulmonary hypertension, and associated disorders were analyzed. Atorvastatin substantially decreased TNF-α (WMD: − 0.20 pg/mL; 95% CI − 0.28 to − 0.11), LDL cholesterol (WMD: − 21.48 mg/dL; 95% CI − 30.82 to − 12.14), and TC (WMD: − 15.24 mg/dL; 95% CI − 28.28 to − 2.20), while improving 6MWD (WMD: 0.71; 95% CI 0.24 to 1.17) and FEF25-75 in COPD subgroups. Evening peak expiratory flow (PEF) was considerably lower (WMD: − 8.72; 95% CI − 14.96 to − 2.47), indicating worsening in airway airflow throughout the evening. There were no significant overall effects for CRP, IL-6, triglycerides, HDL, FEV1, FVC, or oxygen saturation.

**Conclusions:**

Atorvastatin demonstrates anti-inflammatory and lipid-lowering efficacy in pulmonary disease patients, with mild functional respiratory benefits and modest improvements in physical performance. Additional large-scale studies are needed to validate clinical benefits and effective treatment methods.

**Supplementary Information:**

The online version contains supplementary material available at 10.1186/s40001-025-03782-y.

## Introduction

Lung diseases pose a significant global health burden and encompass diverse disorders such as infectious lung diseases (e.g., tuberculosis) [1], mucous-obstructive respiratory diseases (e.g., COPD) [2], and interstitial lung diseases (ILDs) [3]. COPD, the leading obstructive disease, is characterized by elevated mucus accumulation, impairing airway clearance and potentially leading to fatal exacerbations [4, 5]. Pulmonary tuberculosis (TB) remains the most common infectious lung condition worldwide [6]. Early-stage TB may be asymptomatic or present mild fever, with radiographic imaging aiding diagnosis in more advanced stages [7]. Exposure to allergens, hazardous substances such as asbestos, medications, and underlying health conditions, precipitate many ILDs [3, 8–10]. ILDs often manifest as cough, heartburn, and respiratory failure [9], while recurrent TB presents systemic symptoms such as fatigue and night sweats [7]. Moreover, the SARS-CoV-2 virus, responsible for COVID-19, has affected over 700 million individuals globally by 2024 [11]. Despite advances, diseases like pneumonia and asthma continue to present with suboptimal treatment outcomes.

Complementary therapies have become more popular to avoid aggressive treatments and maintain patients' health. hydroxymethylglutaryl-CoA (HMG-CoA) reductase inhibitors, or statins, have become one of the most popular drug classes worldwide. Six statins—pitavastatin, atorvastatin, rosuvastatin, pravastatin, simvastatin, and fluvastatin—are already on the market and are classified as complementary medications [12]. Statins, especially atorvastatin, have become a cornerstone in cardiovascular disease prevention by inhibiting cholesterol synthesis [13–15]. Beyond lipid-lowering, statins exhibit anti-inflammatory effects via NF-κB inhibition and cytokine modulation, suggesting potential therapeutic applications in respiratory diseases [16, 17].

Because of its longer half-life, greater cytokine inhibition, and lipophilicity, atorvastatin has been chosen as a statin candidate for pulmonary function benefits [18–20]. According to a 2016 study on mouse models of bronchial asthma, atorvastatin administration may be useful in reducing respiratory symptoms in mice by inhibiting ovalbumen-induced airway remodeling [21]. To assess the efficacy of these medications in treating lung conditions, numerous RCTs have been carried out on human subjects. Hothersall demonstrated that the use of atorvastatin in conjunction with an anti-asthma medication improved FEV1 both before and after the administration of salbutamol; that morning and evening peak expiratory flow (PEF) values also showed relative improvement; that blood lipids such as HDL, LDL, TG, and certain inflammatory factors in the blood, such as IL-1, were significantly reduced. additionally, the use of this combination can significantly raise bilirubin levels, and the effect of this drug combination on liver function is significant, mediated by increases in blood ALT and AST levels [22]. To date, no comprehensive meta-analysis has synthesized RCT data specifically assessing atorvastatin's impact on inflammatory biomarkers, lipid profile, liver enzymes, and pulmonary function in lung disease patients. This study aims to systematically review and quantitatively analyze RCTs evaluating atorvastatin's effects in patients with pulmonary diseases, elucidating its potential benefits and safety profile.

## Method

### Search strategy

The Preferred Reporting Items for Systematic Reviews and Meta-Analyses Extension for Scoping Reviews (PRISMA-ScR) chart was used as a guide for study selection. The protocol for the current systematic review and meta-analysis was filed in the International Prospective Register of Systematic Reviews (PROSPERO) under the ID number CRD420251148826 to ensure transparency and conformity with acknowledged methodological standards. A comprehensive literature search was conducted up to March 2025 using multiple bibliographic databases, including PubMed/MEDLINE, Embase, the Cochrane Central Register of Controlled Trials (CENTRAL), Scopus, Web of Science, and Google Scholar (as a supplementary source). A professional medical librarian assisted in the development of comprehensive search techniques that included relevant keywords, MeSH phrases, and Boolean operators. Supplementary Materials 1 contains comprehensive search strings for all databases, as well as the date of the last search and the limitations applied. To ensure that no relevant articles were overlooked, we also reviewed the reference lists of every relevant review study.

### Literature inclusion and exclusion criteria

Articles that fulfilled the following inclusion criteria were included: (1) RCTs with parallel or crossover design evaluating atorvastatin in pulmonary disease patients; (2) patients with pulmonary diseases and compared the effects of atorvastatin treatment against placebo or standard care control or no intervention groups; (3) Studies reporting biomarker measurements (such as lipid profile or inflammatory factors) before and after intervention; (4) The experimental interventions comprised atorvastatin monotherapy or atorvastatin combined with other pharmaceutical regimens included corticosteroids, bronchodilators (beta-2 agonists and/or anticholinergics), and/or additional lipid-lowering agents, primarily ezetimibe, depending on trial protocols; (5) Studies published in the English language. The articles were excluded if they were: (1) Observational studies, case series, animal/in vitro studies, or non-randomized trials; (2) Trials comparing statins with other medications rather than placebo; (3) Studies lacking full texts or requisite data for effect size calculation.

### Data extraction

Two independent reviewers (SB and RN) collected relevant data using a standardized data extraction form designed for the study's purposes. Two reviewers screened the titles and abstracts of the studies and independently extracted data on study characteristics (authors, year, country), participant demographics (sample size, mean age, sex), intervention details (atorvastatin dose, duration), and clinical outcomes (inflammatory markers, lipid profile, liver enzymes, pulmonary function tests) in both control and intervention groups. Discrepancies were resolved by consensus or third reviewer adjudication (MA).

### Data extraction

For continuous outcomes, we preferred to extract mean change from baseline values and their associated standard deviations (SDs) wherever possible, in accordance with Cochrane Handbook guidelines to correct for baseline imbalances and improve statistical power. If change-from-baseline data were not supplied, we calculated final mean values and SDs, taking into account this possible source of heterogeneity. Importantly, co-interventions including non-statin medications—such as corticosteroids, bronchodilators, or other lipid-lowering agents—were documented, along with their dosages and duration when reported. Disagreements amongst reviewers were addressed through discussion. Missing outcome data were thoroughly recorded. We attempted to contact the associated authors for clarification or additional results. Sensitivity analyses were conducted, omitting trials with missing or imputed data, to determine the impact on total pooled values and possible effect modification.

### Risk of bias and quality assessment

Study quality and risk of bias were assessed using the Cochrane Risk of Bias tool (Generic) for RCTs, using the structured domain-based methodology recommended in the Cochrane Handbook for Systematic Reviews of Interventions to evaluate sequence generation, allocation concealment, blinding, incomplete outcome data, selective reporting, and other biases. A pair of independent reviewers assessed the probability of each potential bias. Any disagreements were resolved by discussion. For outcomes with fewer than ten studies, possible publication bias was evaluated indirectly using search comprehensiveness and sensitivity analyses that excluded smaller or lower-quality studies. These procedures are intended to reduce bias and increase the validity of the synthesized evidence.

The GRADE (Grading of Recommendations, Assessment, Development, and Evaluation) methodology was used to determine the certainty of evidence for important outcomes. This entailed evaluating the body of evidence based on the risk of bias, inconsistency, indirectness, imprecision, and publication bias using existing GRADE principles. The quality of evidence for each outcome was classified as high, moderate, low, or very low.

### Statistical analysis

Pooled effect sizes were calculated as weighted mean differences (WMDs) with 95% confidence intervals (CIs) for continuous outcomes. Random-effects meta-analyses were performed to account for between-study heterogeneity. We utilized mean change from baseline data where available; when only final values were supplied, we included them with caution and marked them in sensitivity analyses. For studies that did not give SDs of mean changes, we used the Cochrane Handbook approach to impute missing SDs based on baseline and final SDs and an estimated correlation coefficient (default r = 0.5), allowing for fuller study inclusion. Sensitivity analyses were conducted to determine the effect of imputed SDs on pooled estimates. Heterogeneity was assessed with Cochran's Q test and quantified by the I^2^ statistic, with values of 25%, 50%, and 75% representing low, moderate, and high heterogeneity, respectively. Subgroup analyses evaluated effects by pulmonary disease type (infectious, asthma, COPD, others), atorvastatin dose (20 > vs. ≤ 20 mg/day), treatment duration (≤ 30 days, > 30 days), and supplement type (atorvastatin alone vs. combined with other therapies). Publication bias was assessed statistically using Egger’s regression test and Begg’s rank correlation test, with *p* < 0.05 indicating possible bias. All analyses were performed using Stata version 17.

## Result

### Study selection and characteristics

**Fig. 1:**
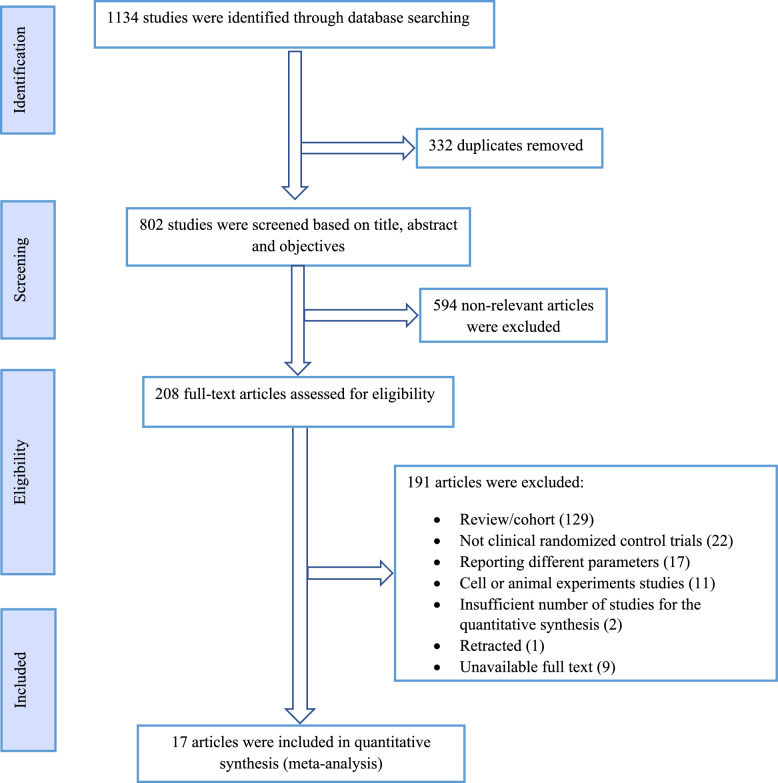
PRISMA flow diagram detailing the study selection process for the meta-analysis

A total of 1134 studies were identified in several database searches. After duplicate removal, 802 were screened for further evaluation. After full-text assessments, finally,17 studies with 22 trials involving 1344 participants were included in the current meta-analysis. The flow diagram of study selection is shown in Fig. 1.

All of the studies were RCTs published between 2008 and 2022. Sample sizes varied widely (17 to 882 patients), the dosages of these medicines varied from 10 to 40 mg/d, and the durations of intervention were between 6 to 180 days. Studies assessed a broad spectrum of pulmonary conditions, including asthma, chronic obstructive pulmonary disease (COPD), pulmonary hypertension, COVID-19, community-acquired pneumonia, obstructive sleep apnea syndrome, and chronic bronchitis. Among reported outcomes were inflammatory markers such as C-reactive protein (CRP, 11 effect sizes from 10 studies), interleukin-6 (IL-6, 6 effect sizes from 5 studies), and tumor necrosis factor-alpha (TNF-α, 3 studies). Pulmonary function indices included forced expiratory volume in 1 s (FEV1, 9 effect sizes from 7 studies), FEV1/FVC ratio (3 effect sizes from 2 studies), morning and evening peak expiratory flow (PEF, 4 effect sizes each from 2 studies), oxygen saturation (4 effect sizes from 3 studies), and forced vital capacity (FVC, 4 effect sizes from 3 studies). Liver enzymes ALT and AST were reported in 3 studies each. lipid profile included total cholesterol (TC, 7 studies), triglycerides (TG, 6 studies), high-density lipoprotein (HDL, 6 studies), and low-density lipoprotein (LDL, 8 studies). The characteristics of the included studies are summarized in Table 1.

**Table 1:** Characteristics of included RCTs evaluating atorvastatin in pulmonary disease patients

Author, year	country	study design	Population target	Intervention	Total subjects	Dose (mg/day)	duration (Days)	Biomarkers
Davoodi, 2021 [23]	Iran	DB RCT Parallel	COVID-19	Atorvastatin + lopinavir/ritonavir	40	40	6	O2Sat/CRP
Mehrabi (a), 2021 [24]	Iran	TB PC RCT	Asthma	Atorvastatin	80	40	28	FEV1, FVC, FEV1/FVC, TC, HDL, LDL, AST, ALT, TG, RV/TLC
Mehrabi (b), 2021 [24]	Iran	TB PC RCT	Asthma	Atorvastatin	80	40	56	FEV1, FVC, FEV1/FVC, TC, HDL, LDL, AST, ALT, TG, RV/TLC
Hothersall (a), 2008 [25]	Scotland	RDB Crossover	Asthma	Atorvastatin, anti-asthma	54	40	56	TG, TG, AST, HDL, ALT, Morning PEF, Evening PEF, FEV1/FVC ratio, IL-6, TNF-α, CRP, FEV1, FVC, FEF 25-75
Hothersall (b), 2008 [25]	Scotland	RDB Crossover	Asthma	Atorvastatin, anti-asthma	54	40	28	Morning PEF, Evening PEF
Hothersall (c), 2008 [25]	Scotland	RDB Crossover	Asthma	Atorvastatin, anti-asthma	54	40	14	Morning PEF, Evening PEF
Ghati (a), 2022 [26]	India	SC Pros 4-arm Parallel OL RCT	COVID-19	Atorvastatin	440	40	10	CRP, TNF-α, IL-6, TG, TC, HDL, AST, ALT
Ghati (b), 2022 [26]	India	SC Pros 4-arm Parallel OL RCT	COVID-19	Atorvastatin, Aspirin	440	40/75	10	CRP, TNF-α, IL-6, TG, TC, HDL, AST, ALT
Momeni, 2021 [27]	Iran	RDB PC Trial	Chronic bronchitis	Atorvastatin	78	40	84	hs-CRP, LDL, TNF-α, IL-6
Moini, 2012 [28]	Iran	DB RCT	Persistent asthma	Atorvastatin	57	40	56	FEV1%, FVC, TC, LDL-C, HDL-C, TG
Bisht, 2017 [29]	India	R CT Open-label	COPD	Atorvastatin, COPD	60	20	84	FEV1%, LDL, hs-CRP
Mohamed, 2019 [30]	Egypt	R Pros Interventional	Pneumonia	Atorvastatin, antibiotics	47	40	7	CRP
Hothersall, 2008 [31]	UK	RDB Crossover	Asthma	Atorvastatin	54	40	56	Morning PEF, Evening PEF, TNF-a, hs-CRP, TNF-a, IL-6, FEV1
Malek Mohammad, 2012 [32]	Iran	RDB PC CT	Bronchial Hyper-responsivity	Atorvastatin	22	20	28	FEV1
Moosavi, 2013 [33]	Iran	TB RCT Parallel	Systolic pulmonary arterial hypertension	Atorvastatin	45	40	180	FEV1, HDL, AST, ALT, FEV1/FVC, FVC
Ghobadi (a), 2013 [34]	Iran	RDB Controlled Trial	COPD	Atorvastatin, COPD	45	40	28	SPO2/ 6MWD, FEV1, FEF 25-75
Ghobadi (b), 2013 [34]	Iran	RDB Controlled Trial	COPD	Atorvastatin, COPD	45	40	63	FEV1, 6MWT, TC, HDL, TG, LDL, hs CRP, FEF 25-75
Mroz, 2015 [35]	Poland	RSB Controlled Parallel Pilot	COPD	Atorvastatin, COPD	17	40	84	FEV1, 6MWT, TC, HDL, TG, LDL, hs CRP, RV/TLC
Zeng, 2012 [36]	China	RDB PC Trial	Pulmonary hypertension patients	Atorvastatin	220	10	168	O2Sat / CRP
Arian, 2018 [37]	Iran	RCT	COPD	Atorvastatin, COPD	34	40	168	IL‑6, hs‑CRP
Joyeux-Faure, 2014 [38]	France	MC RDB RCT Parallel	Obstructive Sleep Apnea Syndrome	Atorvastatin	51	40	84	Total cholesterol, LDL, HDL, Triglycerides, hs-CRP
Mortazavi Moghadam, 2016 [39]	Iran	DB RCT	COPD	Atorvastatin	34	40	180	O2.sat

*O2Sat* oxygen saturation, *CRP* C-reactive protein, *FEV1* forced expiratory volume in one second, *FVC* forced vital capacity, *FEV1/FVC* ratio of FEV1 to FVC, *TC* total cholesterol, *HDL* high-density lipoprotein, *LDL* low-density lipoprotein, *AST* aspartate aminotransferase, *ALT* alanine aminotransferase, *TG* triglycerides, *RV/TLC* residual volume to total lung capacity ratio, *PEF* peak expiratory flow, *IL-6* interleukin-6, *TNF-α* tumor necrosis factor-alpha, *hs-CRP* high-sensitivity C-reactive protein, *6MWD* six-minute walk distance, *6MWT* six-minute walk test, *DB* double-blind, *TB* triple-blind, *PC* placebo-controlled, *RCT* randomized controlled trial, *RDB* randomized double-blind, *SC* single-center, *Pros* prospective, *OL* open-label, *MC* multicenter, *RSB* randomized single blind

### Effect of atorvastatin on inflammatory markers

**Fig. 2:**
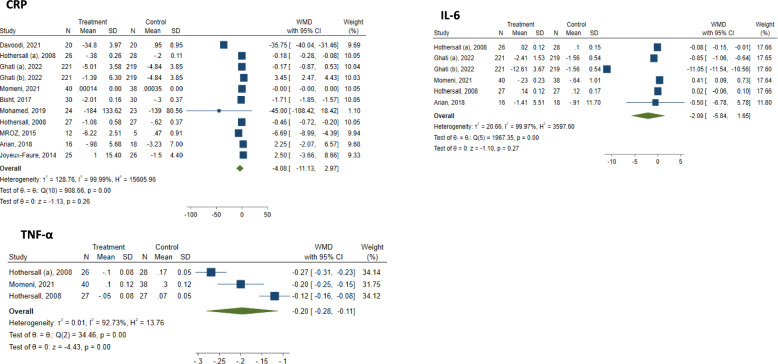
Forest plots of inflammatory biomarkers, including CRP, IL-6, and TNF-α, changes following atorvastatin treatment

Pooling studies revealed a statistically significant reduction in TNF-α serum levels with atorvastatin therapy (WMD: − 0.20 pg/mL; 95% CI − 0.28 to − 0.11; I^2^ = 92.73%). However, no significant effect was found on IL-6 (WMD: − 2.09 pg/mL; 95% CI − 5.84 to 1.65; I^2^ = 99.97%) and CRP levels (WMD: − 4.08 mg/L; 95% CI − 11.13 to 2.97; I^2^ = 99.99%) (Fig. 2).

### Effect of atorvastatin on lipid profile

**Fig. 3:**
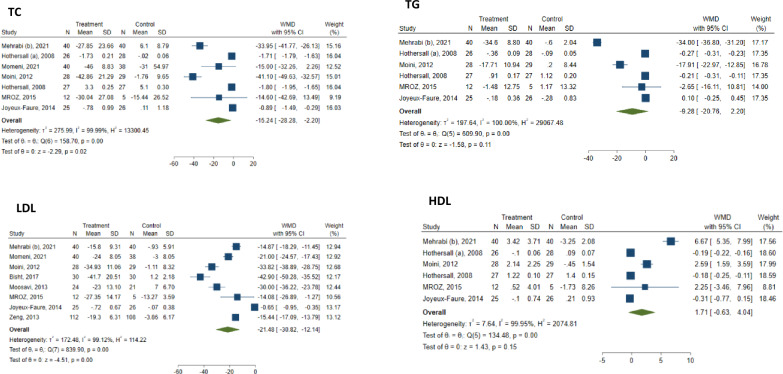
Forest plots of lipid profile, including TC, TG, LDL, and HDL changes following atorvastatin treatment

Meta-analysis of 8 studies showed a robust and statistically significant reduction in LDL (WMD: − 21.48 mg/dL; 95% CI − 30.82 to − 12.14; I^2^ = 99.12%) and TC (WMD: − 15.24 mg/dL; 95% CI − 28.28 to − 2.20; I^2^ = 99.99%). However, no significant overall change was observed in HDL (WMD: 1.71 mg/dL; 95% CI − 0.63 to 4.04; I^2^ = 99.95%) and TG (WMD: − 9.28 mg/dL; 95% CI − 20.76 to 2.20; I^2^ = 100%) (Fig. 3).

### Effect of atorvastatin on liver enzymes

**Fig. 4:**
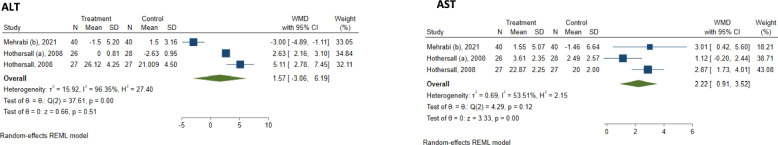
Forest plots of liver enzymes, including ALT and AST changes following atorvastatin treatment

Three studies assessed the impact of statins on liver enzymes like AST and ALT levels. The results showed that a significant increase in AST after atorvastatin administration (WMD: 2.22, 95%CI 0.91 to 3.52; I^2^ = 53.51%) was seen, but no significant difference was found in ALT levels after atorvastatin administration (WMD, 1.57; 95% CI − 3.06 to 6.19; I^2^ = 96.35%) (Fig. 4).

### Subgroup Analysis results

To analyze the significant heterogeneity in primary outcomes (I^2^ frequently > 90%), we conducted subgroup analyses including key covariates: disease type (infectious, asthma, COPD, others), atorvastatin dose (≤ 20 mg/day vs. > 20 mg/day), and treatment duration (≤ 30 days vs. > 30 days). Meta-regressions significantly explained some of the variation between studies for these outcomes (*p*-values < 0.05 for most variables; see Supplementary Table S1). When stratified by therapy type, atorvastatin alone significantly decreased TNF-α (WMD: − 0.16; 95% CI − 0.24 to − 0.08; I^2^ = 83%). In addition, Disease subgroup analysis highlighted a prominent reduction in the levels of TNF-α (WMD: − 0.20; 95% CI − 0.34 to − 0.05; I^2^ = 97.10%) and CRP (WMD: − 0.29; 95% CI − 0.56 to − 0.02; I^2^ = 74.13%) in asthma patients. Dose-stratified analyses indicated reductions of LDL at 10–20 mg/day (WMD, -28.92; 95% CI − 55.83 to − 2.02; I2 = 98.02%) and 40 mg/day WMD, − 19.04; 95% CI − 28.99 to − 9.09; I^2^ = 98.10%) of atorvastatin, both statistically significant. using atorvastatin alone significantly reduce TC (WMD: − 18.29; 95% CI − 34.87 to − 1.70; I^2^ = 99.94%). In addition, Combination with anti-COPD medications also produced significant decreases in LDL (WMD: − 28.98; 95% CI − 57.21 to 0.76; I^2^ = 93.15%) and TC (WMD: − 1.71; 95% CI − 1.79 to − 1.63; I^2^ = 0%). Subgroup evaluations reaffirmed the LDL-lowering benefit in asthma (WMD: − 24.25) and COPD (WMD: − 28.98) populations. Notably, combination therapy with anti-asthma or COPD medications led to significant decreases in TG (WMD: − 0.27; 95% CI − 0.31 to − 0.23; I^2^ = 0%), while atorvastatin monotherapy was ineffective. Subgroup analysis based on the type of supplement showed that atorvastatin monotherapy increased the level of AST (WMD: 2.89; 95% CI 1.85 to 3.93; I^2^ = 0%). Detailed subgroup analyses for additional outcomes are provided in Supplementary Table S1.

### Respiratory function tests

**Fig. 5:**
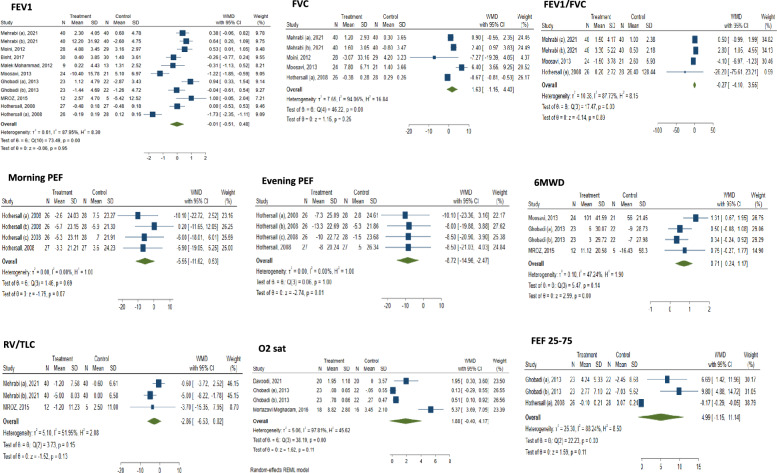
Forest plots of pulmonary functional and clinical outcomes, including Forced Expiratory Volume in 1 s (FEV1) changes, Forced Vital Capacity (FVC) changes, FEV1/FVC ratio, morning Peak Expiratory Flow (PEF), evening PEF, Six-Minute Walk Distance (6MWD), Residual Volume/Total Lung Capacity ratio (RV/TLC), Oxygen saturation (SpO₂), and Forced Expiratory Flow at 25–75% (FEF25-75) following atorvastatin treatment

Pooling data indicated a significant increase in 6-Minute Walk Distance (6MWD) (WMD: 0.71; 95% CI 0.24 to 1.17; I^2^ = 47.24%) and a significant decrease in evening Peak Expiratory Flow (PEF) (WMD: − 8.72; 95% CI − 14.96 to − 2.47; I^2^ = 0%) post-atorvastatin. This improvement in 6MWD persisted when atorvastatin was used in conjunction with other supplements and in COPD patients (WMD: 0.47; 95% CI 0.08 to 0.85; I^2^ = 0%). Evening PEF significantly decreased with atorvastatin combined with anti-asthma medications (WMD: − 8.79 L/min; 95% CI − 15.99 to − 1.59; I^2^ = 0%). Longer duration (> 60 days) was associated with more pronounced 6MWD gains (WMD: 0.80; 95% CI 0.16 to 1.43; I^2^ = 57.63%) and significant improvement in RV/TLC Ratio (WMD: − 4.91; 95% CI − 8.01 to − 1.81; I^2^ = 0%) (Fig. 5), and Evening PEF (WMD: − 9.25; 95% CI − 18.36 to − 0.15; I^2^ = 0%). Interestingly, significant improvement occurred in FEF25–75 (WMD: 8.35; 95% CI 4.75 to 11.95; I^2^ = 0%) in COPD subgroups. Meta-analyses on multiple pulmonary function parameters are summarized in Supplementary Table S2 and Fig. 5.

### Risk of bias assessment and publication bias

**Fig. 6:**
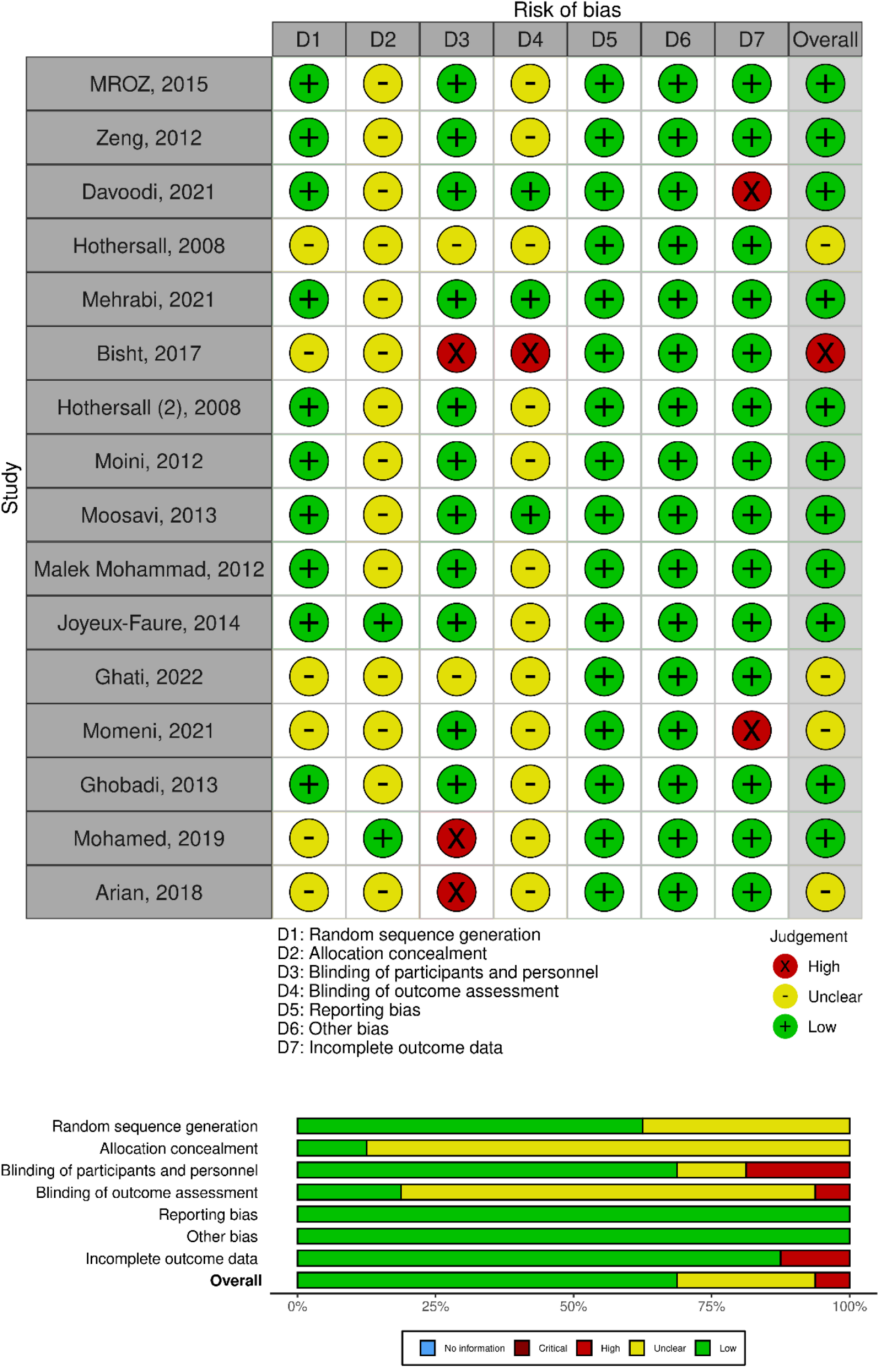
Risk of bias summary across included RCTs using the Cochrane tool, illustrating proportions of low, unclear, and high risk per domain

Figure 6 shows an overview of the risk of bias evaluation across the included studies. The key domains with the highest incidence of unclear risk were allocation concealment and blinding (for both participants and outcome assessors). A small proportion of research showed a low risk of bias, whereas attrition and reporting bias risks were typically low or unclear.

The presence of publication bias was assessed for all primary outcomes using Egger’s linear regression and Begg’s rank correlation tests (Table 2). The findings demonstrated no significant small-study effects or publication bias for most parameters except for O2 (*p* = 0.019) and FEF25-75 (*p* < 0.001) in Egger’s test. As a result, data on oxygen saturation and FEF25-75 should be regarded with caution, and further validation through appropriately powered studies is required. Because of the selective selection of studies showing positive outcomes, publication bias may cause treatment effects to be overestimated. Sensitivity analyses, including the exclusion of smaller studies, resulted in lower effect estimates and increased uncertainty, highlighting the potential influence of bias on these parameters (Supplementary Figure S1).

**Table 2:** Publication Bias Assessment Using Egger's Linear Regression and Begg's Rank Correlation Tests

Outcome	Egger’s Test (p-value)	Begg’s Test (p-value)
CRP	0.147	1
TNF-α	0.978	1
IL-6	0.866	0.26
TC	0.359	0.548
TG	0.847	1
HDL	0.443	0.133
LDL	0.233	1
AST	0.72	1
ALT	0.996	1
Morning PEF	0.289	0.089
Evening PEF	0.816	0.089
6MWD	0.779	0.308
FEV1	0.92	0.436
FEF25-75	< 0.001	1
FVC	0.468	0.807
FEV1/FVC	0.208	0.734
RV/TLC	0.879	1
Oxygen Saturation (SpO2)	0.019	0.308

### Evaluation of certainty

The GRADE method was used to assess the certainty of evidence for each outcome carefully. The evidence was categorized into four levels of quality: high, moderate, low, and very low [40]. This evaluation was carried out by a single reviewer (MA). The downgrading was mostly caused by inconsistency and imprecision (Table 3).

**Table 3:** GRADE profile of atorvastatin effects on inflammatory markers, lipid profile, liver enzymes, pulmonary function, and physical performance in pulmonary disease patients

Outcomes	Risk of bias	Inconsistency	Indirectness	Imprecision	Publication bias	Effect size (95% CI)	Quality of evidence
TNF-α	Not serious	Very serious[a]	Not serious	Serious	Not Serious	− 0.20 [− 0.28 to − 0.11]	⊕ ⊕ ⊝ ⊝ Low
IL-6	Not serious	Very serious[a]	Not serious	Serious	Not Serious	− 2.09 [− 5.84 to 1.65]	⊕ ⊕ ⊝ ⊝ Low
CRP	Not serious	Very serious[a]	Not serious	Serious	Not Serious	− 4.08 [− 11.13 to 2.97]	⊕ ⊕ ⊝ ⊝ Low
LDL	Not serious	Very serious[a]	Not serious	Serious	Not serious	− 21.48 [− 30.82 to − 12.14]	⊕ ⊕ ⊝ ⊝ Low
TC	Not serious	Very serious[a]	Not serious	Serious	Not serious	− 15.24 [− 28.28 to − 2.20]	⊕ ⊕ ⊝ ⊝ Low
HDL	Not serious	Very serious[a]	Not serious	Serious	Not serious	1.71 [− 0.63 to 4.04]	⊕ ⊕ ⊝ ⊝ Low
TG	Not serious	Very serious[a]	Not serious	Not serious	Not serious	− 9.28 [− 20.76 to 2.20]	⊕ ⊕ ⊕ ⊝ Moderate
AST	Not serious	Not serious	Not serious	Not serious	Not serious	2.22 [0.91 to 3.52]	⊕ ⊕ ⊕ ⊕ High
ALT	Not serious	Very serious	Not serious	Serious	Not serious	1.57 [− 3.06 to 6.19]	⊕ ⊕ ⊝ ⊝ Low
FEV1	Not serious	Serious	Not serious	Serious	Not serious	− 0.01 [− 0.51 to 0.48]	⊕ ⊕ ⊝ ⊝ Low
FVC	Not serious	Serious	Not serious	Serious	Not serious	1.63 [− 1.15 to 4.40]	⊕ ⊕ ⊝ ⊝ Low
FEV1/FVC	Not serious	Serious	Not serious	Serious	Not serious	− 0.27 [− 4.10 to 3.55]	⊕ ⊕ ⊝ ⊝ Low
RVTLC	Not serious	Not serious	Not serious	Not serious	Not serious	− 2.86 [− 6.53 to 0.82]	⊕ ⊕ ⊕ ⊕ High
Morning PEF	Not serious	Not serious	Not serious	Not serious	Not serious	− 5.55 [− 11.62 to 0.53]	⊕ ⊕ ⊕ ⊕ High
Evening PEF	Not serious	Not serious	Not serious	Not serious	Not serious	− 8.72 [− 14.96 to − 2.47]	⊕ ⊕ ⊕ ⊕ High
Oxygen Saturation	Not serious	Serious	Not serious	Not serious	Serious [b]	1.88 [− 0.4 to 4.17]	⊕ ⊕ ⊝ ⊝ Low
6-Minute Walk Distance	Not serious	Not serious	Not serious	Not serious	Not serious	0.71 [0.24 to 1.17]	⊕ ⊕ ⊕ ⊕ High
FEF25-75	Not serious	Not serious	Not serious	Not serious	Serious [b]	4.99 [-1.15 to 11.14]	⊕ ⊕ ⊝ ⊝ Low

a Graded very serious due to the inconsistency (I^2^ > 75%); not serious when consistency is high (I^2^ < 50%); and serious when inconsistency is moderate (50% ≤ I^2^ ≤ 75%)

b Significant publication bias was noted for oxygen saturation (P = 0.019) and FEF25-75 (P < 0.001) based on Egger’s test. No significant bias was detected for other parameters

## Discussion

This meta-analysis comprehensively examined the effects of atorvastatin on inflammatory markers, lipid profile, liver enzymes, pulmonary function, and physical performance across a diverse set of 17 RCTs, involving patients with different pulmonary diseases, including COVID-19, community-acquired pneumonia, pulmonary hypertension, COPD, asthma, and chronic bronchitis. The analysis highlights that atorvastatin, primarily known for its lipid-lowering effects, also exerts substantial immunomodulatory and anti-inflammatory actions that may contribute to therapeutic benefits in pulmonary diseases. Subgroup analyses by disease type, dosage, and duration show a generally similar pattern of effect, with higher doses and longer therapy durations associated with better biochemical and functional results, notably in the asthma and COPD groups. These findings support and expand on previous evidence of statins' pleiotropic effects in pulmonary disease modulation [41]. Contrasting findings in the literature emphasize the continuous importance of well-planned trials to develop uniform methods and identify groups most likely to benefit.

Furthermore, the current comprehensive analysis suggests that atorvastatin, both alone and in combination with corticosteroids, bronchodilators, and ezetimibe, may have a positive effect on inflammatory markers and lipid profile in a variety of pulmonary diseases. Combination treatment appears to boost these effects, in line with cardiovascular research findings, and better results with statin-ezetimibe regimens [42]. However, variability and poor data in certain subgroups prevent clear findings. We prioritize narrative synthesis over pooled effect estimation for outcomes with I^2^ > 90% due to extreme heterogeneity and variability in study designs and populations. This approach provides a transparent portrait of effect directionality and variability, avoiding potentially misleading aggregate summaries. These findings highlight the necessity for future well-powered, disease-specific RCTs with standardized treatments to create more uniform evidence and reliable impact estimates.

The anti-inflammatory effect of atorvastatin is evident in several trials. Specifically, a significant reduction in TNF-α levels was observed, particularly in patients with asthma, which supports the hypothesis that statins may modulate systemic inflammation in chronic respiratory diseases. Interestingly, while IL-6 and CRP levels did not show significant reductions overall, the subgroup of asthma patients exhibited a significant decrease in CRP, suggesting a potentially disease-specific anti-inflammatory response. Asthma patients showed greater decreases in CRP and TNF-α compared to infection or COPD groups, indicating that disease type has a substantial impact on treatment effects for inflammatory markers. These findings are consistent with previous research showing that statins can attenuate chronic inflammation through multiple pathways, including NF-κB inhibition and modulation of immune cell function. A recent double-blind randomized trial corroborated these findings by demonstrating a significant decline in CRP and reduced hospitalization duration following a 5-day course of atorvastatin [43].

Nevertheless, the clinical translation of these biochemical effects remains inconsistent. No significant improvements were detected in oxygen saturation, ICU admission rates, mechanical ventilation requirements, or the need for adjunctive therapies such as interferons or immunoglobulins [43]. This discrepancy suggests that while atorvastatin may modulate inflammation, this alone might not be sufficient to alter major clinical endpoints in the acute management of pulmonary diseases. Animal studies have demonstrated reduced infiltration of inflammatory cells and airway hyper-responsiveness with statin administration [21, 44]. These effects may be partially mediated by atorvastatin’s ability to upregulate ACE2 expression, counteracting its SARS-CoV-2-induced downregulation, and by suppressing the NF-κB pathway, thus reducing the secretion of proinflammatory cytokines like IL-6 and CRP [45]. In contrast, Moini et al. observed reduced eosinophil counts in both the placebo and intervention groups, suggesting the presence of confounding factors such as natural disease fluctuation or concomitant therapies. The differential response may stem from variations in the Th1/Th2 lymphocyte balance, with the inhibitory effect of statins on Th2 cells, which play a dominant role in asthma pathogenesis, remaining uncertain [46]. Hothersall et al. demonstrated that atorvastatin significantly reduced sputum macrophage counts, LTB4, and CRP levels in patients with atopic asthma; however, this did not translate into short-term clinical improvements [31]. Reviews have indicated that atorvastatin reduces airway inflammation in patients with asthma, but does not improve lung function; however, the investigated inflammatory markers vary among the studies [47]. Other studies echoed similar patterns. For example, Ghati et al. found that a combination of aspirin and atorvastatin significantly reduced serum IL-6 [26], while experimental models showed suppression of cytokine production and redox markers [48]. Atorvastatin appears to stabilize MYD88 levels under inflammatory stress, potentially reducing the intensity of the immune response [49]. Additionally, statins have been shown to upregulate ACE2 via epigenetic mechanisms, which may be protective against SARS-CoV-2-mediated lung injury [50, 51]. Several observational studies suggest that prior statin use is associated with reduced mortality in hospitalized COVID-19 patients [26]. The proposed mechanisms include uncoupling of oxidative phosphorylation in mitochondria [52], nitric oxide induction [53], and NF-κB inhibition [54]. However, Momeni et al. found no significant reductions in inflammatory markers (IL-6, TNF-α, hs-CRP, or procalcitonin) in the intervention group, while the placebo group showed a significant increase in TNF-α, suggesting a protective, though not strongly therapeutic, effect of atorvastatin [27]. The effectiveness of atorvastatin also varies depending on the formulation. For instance, Bisht et al. found that lipophilic atorvastatin (20 mg/day) did not affect lung function or quality of life in COPD patients, though it significantly reduced hs-CRP [55]. Evidence suggests a dose–response relationship in the anti-inflammatory effect of atorvastatin, ranging from 10 to 80 mg [56], with 20 mg being both effective and well-tolerated [57]. Mohamed’s study, focusing on pneumonia patients, revealed a significant reduction in CRP and hospital stay with atorvastatin-antibiotic co-therapy, a finding confirmed by a large-scale study conducted by Mortensen et al. [30, 58]. The anti-inflammatory mechanism of statins is attributed to inhibition of the mevalonate pathway, which blocks prenylation of small GTPases like Ras. This results in the suppression of downstream effectors such as NF-κB and a subsequent reduction in cytokine production [59].

In clinical settings, inhaled corticosteroids used concurrently with atorvastatin may enhance anti-inflammatory effects, as suggested by reductions in sputum macrophages and leukotrienes without changes in spirometric indices [25]. The benefit of statins may thus lie more in reducing systemic inflammation than improving lung function directly. Previous studies showed decreased hs-CRP levels in COPD patients on statin therapy [60] and lower mortality rates [61]. Genomic and transcriptomic studies further support atorvastatin’s immunomodulatory role. Mroz et al. observed significant downregulation of genes involved in immune signaling, leukocyte activation, focal adhesion, ECM interaction, and NK cell-mediated cytotoxicity. Another study also noted increased expression of EGR1 and downregulation of cancer-related pathways, suggesting possible protective effects in comorbid COPD-lung cancer settings [35, 62]. The anti-inflammatory mechanisms of other statins and their effects on the lung have been studied in vitro. Simvastatin has demonstrated comparable anti-inflammatory effects, such as inhibiting matrix metalloproteinase-9 and leukocyte infiltration [63–65]. Thomson’s study revealed reductions in sputum concentrations of multiple cytokines and growth factors with atorvastatin, and improvements in quality of life were associated with reductions in inflammatory biomarkers [66]. The core pharmacological action of statins involves competitive inhibition of HMG-CoA reductase, reducing cholesterol synthesis and producing immunomodulatory effects [67, 68]. They also upregulate ACE2 in pulmonary tissues, potentially mitigating ARDS severity without promoting viral entry [69, 70]. However, statins’ muscle-related side effects, including myositis and rhabdomyolysis, are well-documented, particularly when co-administered with CYP3A inhibitors like ritonavir or lopinavir [71]. Some studies, including those by Arian and Young et al., reported reductions in IL-6 and hs-CRP that were not statistically significant [37, 72]. IL-6 plays a pivotal role in pulmonary hypertension associated with COPD [73], and in vitro studies show that atorvastatin suppresses IL-6 production in endothelial cells [74]. The modest rise in total antioxidant capacity (TAC) may result from reduced production of coenzyme Q10, which is suppressed by statin therapy [75].

The weighted mean difference of − 0.20 pg/mL in TNF-α is likely a small absolute change with questionable therapeutic consequences, given the absence of recognized MCID guidelines for inflammatory cytokines in respiratory disorders. Similarly, the pooled improvement in six-minute walk distance (0.71 units) is significantly lower than the widely accepted MCID range of 25–35 m for chronic respiratory diseases, indicating minimal discernible effect. The contrast between statistical and clinical significance is crucial for evaluating meta-analytic data from diverse populations. As a result, rather than providing definitive clinical evidence, our findings should be seen as indicators of possible molecular consequences that require additional exploration. To completely describe atorvastatin's therapeutic impact in pulmonary diseases, we recommend that future research use validated MCIDs and patient-centered outcome measures.

The findings of this meta-analysis indicate that atorvastatin has a favorable impact on certain lipid profile parameters in patients with chronic pulmonary diseases. Specifically, a significant reduction in LDL and TC levels was observed, while HDL and TG levels remained largely unchanged. Subgroup analyses demonstrated that when atorvastatin was administered alongside medications for asthma or COPD, greater improvements in LDL and TG levels were achieved. Moreover, the LDL-lowering effect of atorvastatin appeared to be dose-dependent across 10, 20, and 40 mg dosages. These observations suggest that atorvastatin may contribute to improved lipid metabolism, thereby potentially reducing cardiovascular risk in these patient populations. Supporting this, the study by Mehrabi et al. reported a significant reduction in serum levels of TC, LDL, and TG, as well as a significant change in HDL in the intervention group [24]. Similarly, Momeni et al. concluded that the benefits of intensive statin therapy are likely linked to reductions in both LDL and CRP levels [27]. In Hothersall’s study, atorvastatin led to significant reductions in serum lipid concentrations, although an increase in cholesterol and HDL-cholesterol was also noted [25]. Moini et al. further highlighted the efficacy of atorvastatin, showing significant differences in LDL-C and TC levels between intervention and control groups, implying good adherence to treatment [46]. Zeng et al. demonstrated that atorvastatin 10 mg/day significantly lowered cholesterol levels in patients with pulmonary hypertension [36]. These lipid-lowering effects may be partially attributed to atorvastatin’s capacity to enhance endothelial nitric oxide synthase expression and inhibit cell proliferation while promoting apoptosis, as reported in animal models [76]. Despite these effects, considerable residual heterogeneity persisted, indicating the complicated interaction of other unmeasured clinical and methodological variables among the included studies. Given the small number of studies in multiple subgroups and the weak statistical power, these meta-regression findings should be viewed as exploratory and hypothesis-generating rather than confirming.

Regarding hepatic safety, a mild and non-significant increase in ALT was recorded, while AST levels significantly increased, particularly in monotherapy settings. Due to the limited number of studies and notable heterogeneity, these findings warrant cautious interpretation. Nevertheless, they suggest that atorvastatin may induce mild hepatic changes, necessitating careful monitoring in clinical contexts. The Hothersall study also observed a modest yet significant elevation in bilirubin, AST, and ALT levels [25].

Our meta-analysis found that atorvastatin therapy caused a slight mean increase in AST levels (WMD: 2.22 U/L), which is unlikely to be clinically meaningful for the majority of patients. This moderate increase is consistent with statins' recognized safety profile, which is well-documented to induce temporary and asymptomatic increases in liver enzymes rather than overt liver impairment or hepatotoxicity [77]. Indeed, the majority of patients tolerate statins without experiencing hepatic side effects, and baseline liver enzyme evaluation paired with periodic follow-up is considered routine therapeutic practice rather than an indicator of a new safety risk. However, the substantial heterogeneity reported in alanine ALT level (I^2^ = 96.35%) suggests significant variability among investigations. This heterogeneity may be due to differences in research demographics, comorbidities, statin doses, treatment duration, and test techniques, emphasizing the significance of individualized medical assessment when starting and monitoring statin medication.

Our recommendation for careful tracking should be interpreted in light of current class-effect warnings given by regulatory bodies, which urge liver function testing before and throughout statin therapy to detect rare occurrences of idiosyncratic liver damage. This meta-analysis does not reveal a novel signal of hepatotoxicity, but rather strengthens previously recognized clinical warnings. Real-world evidence and subsequent meta-analyses corroborate statins' favorable hepatic safety profile, especially among patients with underlying liver diseases such as nonalcoholic fatty liver disease (NAFLD) [78]. Furthermore, several studies have found that statins may protect against the progression of liver disease [79]. In conclusion, whereas minor variations in liver enzymes may occur after atorvastatin medication, these changes are often not clinically significant. Our findings support existing guidelines advising baseline and periodic liver enzyme monitoring without excess concerns about hepatotoxicity in standard clinical usage.

In terms of pulmonary function, no significant changes were observed in common indices, including FEV1, FVC, FEV1/FVC, RV/TLC, or oxygen saturation. However, a notable reduction in nocturnal PEF was documented, particularly when atorvastatin was used concurrently with asthma medications over 56 days, indicating potential relief from nocturnal respiratory symptoms. Additionally, in COPD patients, a significant increase in FEF25–75 after treatment suggested better small airway performance. Hothersall et al. reported no significant difference in morning PEF after 8 weeks of atorvastatin therapy, with only FVC being significantly lower in the atorvastatin group [25]. In some studies, short-term treatment with atorvastatin did not change lung function but improved quality of life in smokers with mild to moderate asthma [80]. Retrospective studies have suggested potential benefits of statins in COPD outcomes, including exacerbation frequency, lung function, and mortality rates [35, 81]. Mehrabi et al. and Fanak et al. found no spirometric improvement in asthmatic patients between intervention and placebo groups, although some reports, such as Fanak’s, noted a significant rise in FVC, while Mehrabi found only a reduction in TLC and RV [24, 82]. Differences among studies may relate to varying dosages and treatment durations. In one trial with 62 asthmatic patients, both intervention and placebo groups showed FEV1 improvement, but no intergroup differences in spirometric outcomes were noted after 8 weeks of atorvastatin 40 mg/day [46]. Another study found non-significant changes in TLC count, pneumonia score, and mortality within 30 days of pneumonia diagnosis [30]. Malek’s findings also showed no significant spirometric changes after one month of 20 mg/day atorvastatin in normolipidemic patients with bronchial hyperresponsiveness [83]. Ghobadi’s study noted improved quality of life via CAT and SGRQ scores in COPD patients, without significant changes in FEV1 or SpO₂, suggesting that PFTs may not fully reflect systemic inflammatory responses in pulmonary diseases [34]. Braganza et al. also reported improved asthma quality of life after short-term atorvastatin use in smokers with mild asthma, despite unchanged lung function [80]. Mortazavi et al. found increased oxygen saturation and decreased WBC count with 40 mg/day atorvastatin over six months, while other parameters like pH and PCO₂ remained unaffected. Mortality in COPD patients was associated with age, disease duration, hemoglobin, and hematocrit levels [84]. Studies on the use of statins in COVID-19 have yielded mixed results in disease severity, intensive care unit admission rates. These discrepancies may be explained by differences in study design, sample size, statistical methods, and demographic characteristics [43].

As for physical performance, atorvastatin was associated with significant improvements in 6MWD in some studies, particularly when combined with adjunct treatments and over longer durations (> 60 days). These effects are likely attributable to atorvastatin’s anti-inflammatory and hemodynamic properties. Nevertheless, Moosavi et al. reported no statistical differences in 6MWD or other parameters between atorvastatin and placebo in COPD patients with pulmonary hypertension [33]. Similarly, Mroz et al. documented improved SGRQ scores and a trend toward better 6-min walk distance (6MWD) without changes in lung function [35]. According to Zeng et al., atorvastatin reduced pulmonary vascular resistance and improved exercise capacity, although it did not significantly impact 6MWD, cardiopulmonary hemodynamics, or survival over six months [36].

Our meta-analysis examines data for a wide range of respiratory disorders, including asthma, COPD, COVID-19, and others. We are completely aware that grouping these significantly varied disease entities reduces the clinical specificity and significance of any estimated "average" effect size. Pathophysiological variation and diverse therapy responses among these disorders add significant variability in results, reducing the generalizability of pooled estimates. As a result, we underline that our findings should be viewed largely in terms of hypothesis generation. Significant decreases in TNF-α concentrations were detected in asthma subpopulations, while improvements in small airway function (FEF25-75) were mostly reported in COPD patients. These disease-specific results indicate that atorvastatin's therapeutic advantages may be more prominent in certain clinical contexts rather than across all respiratory diseases. Given the small number of studies in some categories and the high heterogeneity, these findings should be regarded with caution. Rather than offering strong evidence for immediate treatment, our findings suggest significant fields for future, more targeted study. To carefully investigate the efficacy and safety of atorvastatin in precisely defined pulmonary disease groups, well-powered randomized controlled studies with established dose regimens are necessary.

Given the large variety of atorvastatin dosages and treatment periods covered in the current meta-analysis, future research should benefit from Model-Based Meta-Analysis (MBMA) methods [85]. MBMA is a sophisticated mathematical approach that advances beyond typical meta-analysis by simulating dose–response and time-course connections with longitudinal data and pharmacologic principles. This technique may integrate several data sources, account for continuous factors such as dosage and duration, and offer more accurate, clinically actionable information on optimal dosing and treatment regimens. Furthermore, MBMA can better address heterogeneity by utilizing mechanistic expertise, allowing for a more accurate assessment of variability across patient groups and research designs. While data constraints precluded MBMA implementation in the current study, if more detailed individual patient or summary-level longitudinal data become available, MBMA presents an option for fine-tuning dosage optimization and customizing atorvastatin treatment in pulmonary diseases. This method is consistent with growing best practices in medication development and evidence synthesis, enabling informed clinical decision-making based on a comprehensive review of existing data.

## Limitations and interpretations of heterogeneity

Our meta-analysis revealed significant statistical heterogeneity across primary outcomes, with I^2^ values above 90% and approaching 100% for measures including CRP, IL-6, and lipid profile. Such data show substantial variation in actual treatment effects among the included trials, implying that pooled effect estimates should be regarded with caution. This variation stems from both clinical and methodological factors. Clinical trials enrolled patients with a wide range of pulmonary diseases, including acute infectious conditions like COVID-19 and pneumonia, as well as chronic inflammatory disorders like asthma and COPD, which differ significantly in disease pathophysiology, severity, inflammatory profiles, and treatment response. Methodological variability was also exacerbated by differences in atorvastatin dosage (10 to 40 mg/day), treatment duration (6 to 180 days), and monotherapy versus combination regimens. To account for these disparities between studies, random-effects meta-analysis models were used when applicable. Nonetheless, the intrinsic diversity of research populations and treatments restricts the clinical generalizability of any single pooled mean impact, highlighting the fact that these findings represent an average across varied settings rather than definite treatment outcomes.

We conducted subgroup and meta-regression studies to investigate potential causes of heterogeneity, and found that disease type, dosage, and treatment duration all contribute to variability but do not entirely account for it. Given the modest number of studies within specific subgroups and residual unexplained heterogeneity, the subgroup results and meta-regression findings should be interpreted as exploratory and hypothesis-generating. As a result, these findings should be viewed with caution and not regarded as conclusive evidence of treatment effect differences.

Second, the risk of bias assessment found that several of the included studies had unclear reporting or possible weaknesses in allocation concealment and blinding. Inadequate allocation concealment may have generated selection biases, but insufficient blinding raises the possibility of performance and detection biases, especially for subjective goals. These variables may have contributed to the observed variability and influenced impact size estimations. As a result, conclusions should be taken with caution, and the confidence of evidence should be reduced to reflect these limitations.

## Conclusion

This meta-analysis suggests that atorvastatin may have beneficial effects in reducing TNF-α, LDL, total cholesterol, and improving respiratory function in specific clinical contexts such as asthma and COPD. However, its effects on other parameters such as FEV1, CRP, IL-6, TG, and HDL remain limited and appear to depend on multiple factors, including disease type, dosage, treatment duration, and concomitant medication use. Consequently, further well-designed clinical trials with larger populations are needed to confirm these findings and elucidate the precise mechanisms of atorvastatin's effects.

## Supplementary Information

Supplementary material 1.

## Data Availability

The data that support the findings of this study are available from the corresponding author upon reasonable request.
