# Supplementary material for: Effects of atorvastatin on inflammatory markers, lipid profile, liver enzymes, and pulmonary function in patients with lung diseases: a systematic review and meta-analysis of randomized controlled trials
Source: Eur J Med Res. 2026 Jan 19;31:111. doi: 10.1186/s40001-025-03782-y (PMC12825185; doi:10.1186/s40001-025-03782-y)
Supplement: Supplementary file 1 — Supplementary material 1. [file 40001_2025_3782_MOESM1_ESM.docx]

**Supplementary Materials 1.** Detailed Search Strategies and Syntax Used Across Multiple Databases for Identifying Randomized Controlled Trials on Atorvastatin in Pulmonary Diseases.

**PubMed Search String**

- Date of Last Search: March 2025
- Search Terms:

("Atorvastatin"[Title/Abstract] OR "HMG-CoA"[Title/Abstract])

AND

("Pulmonary Diseases"[Mesh] OR "lung disease"[Title/Abstract] OR "Pulmonary Hypertension"[Title/Abstract] OR "Pneumothorax"[Title/Abstract] OR "Lung Inflammation"[Title/Abstract] OR "Bacterial infection"[Title/Abstract] OR "Tuberculosis"[Title/Abstract] OR "SARS-CoV"[Title/Abstract] OR "Asthma"[Title/Abstract])

AND

("Inflammation"[Mesh] OR "Inflammatory biomarkers"[Title/Abstract] OR "lipid profile"[Title/Abstract] OR "Liver Enzyme"[Title/Abstract] OR "pulmonary function tests"[Title/Abstract] OR "physical performance"[Title/Abstract])

AND

("Randomized Controlled Trial"[Mesh] OR "clinical trial"[Title/Abstract] OR "trial"[Title/Abstract])

**Embase Search String**

- Date of Last Search: March 2025
- Search Terms (using Emtree and free-text):

('atorvastatin':ti,ab OR 'hmg coa':ti,ab)

AND

('pulmonary disease'/exp OR 'lung disease':ti,ab OR 'pulmonary hypertension':ti,ab OR 'pneumothorax':ti,ab OR 'lung inflammation':ti,ab OR 'bacterial infection':ti,ab OR 'tuberculosis':ti,ab OR 'sars-cov':ti,ab OR 'asthma':ti,ab)

AND

('inflammation'/exp OR 'inflammatory biomarker':ti,ab OR 'lipid profile':ti,ab OR 'liver enzyme':ti,ab OR 'pulmonary function test':ti,ab OR 'physical performance':ti,ab)

AND

('randomized controlled trial'/exp OR 'clinical trial':ti,ab OR 'trial':ti,ab)

**Cochrane Central Register of Controlled Trials (CENTRAL) Search String**

- Date of Last Search: March 2025
- Search Terms:

(Atorvastatin OR HMG-CoA)

AND

(Pulmonary Diseases OR Lung Disease OR Pulmonary Hypertension OR Pneumothorax OR Lung Inflammation OR Bacterial Infection OR Tuberculosis OR SARS-CoV OR Asthma)

AND

(Inflammation OR Inflammatory Biomarkers OR Lipid Profile OR Liver Enzyme OR Pulmonary Function Tests OR Physical Performance)

AND

(Randomized Controlled Trial OR Clinical Trial OR Trial)

**Scopus Search String**

- Date of Last Search: March 2025
- Search Terms:

TITLE-ABS-KEY(atorvastatin OR "hmg coa")

AND

TITLE-ABS-KEY("pulmonary disease" OR "lung disease" OR "pulmonary hypertension" OR pneumothorax OR "lung inflammation" OR "bacterial infection" OR tuberculosis OR "sars-cov" OR asthma)

AND

TITLE-ABS-KEY(inflammation OR "inflammatory biomarker" OR "lipid profile" OR "liver enzyme" OR "pulmonary function test" OR "physical performance")

AND

(LIMIT-TO(DOCTYPE, "ar") AND LIMIT-TO(LANGUAGE, "English"))

**Web of Science Search String**

- Date of Last Search: March 2025
- Search Terms:

TS=(atorvastatin OR "hmg coa")

AND

TS=("pulmonary disease" OR "lung disease" OR "pulmonary hypertension" OR pneumothorax OR "lung inflammation" OR "bacterial infection" OR tuberculosis OR "sars-cov" OR asthma)

AND

TS=(inflammation OR "inflammatory biomarker" OR "lipid profile" OR "liver enzyme" OR "pulmonary function test" OR "physical performance")

AND

Document Types=(Article) AND Languages=(English)

**Google Scholar Search Strategy**

- Date of Last Search: March 2025
- Search Terms:

"atorvastatin" AND ("lung disease" OR "pulmonary disease") AND ("inflammation" OR "lipid profile" OR "liver enzyme" OR "pulmonary function") AND ("randomized controlled trial" OR trial)

**Supplementary Table 1.** Atorvastatin Effects on Biochemical Biomarkers with 95% CIs by Subgroup Analysis

|  | WMD | CI | I^2^ | P |
| --- | --- | --- | --- | --- |
| ALT |  |  |  |  |
| Total | 1.57 | [-3.06, 6.19] | 96.35% | 0.00 |
|  |  |  |  |  |
| Supplement |  |  |  |  |
| Atorvastatin | 1.03 | [-6.92, -8.97] | 96.44% | 0.00 |
| Atorvastatin+ routine medication | 2.63 | [2.16, 3.10] | .% | . |
| AST |  |  |  |  |
| Total | 2.22 | [0.91, 3.52] | 53.51% | 0.12 |
| Supplement |  |  |  |  |
| Atorvastatin+ routine medication | 1.12 | [-0.20, 2.44] | . | . |
| Atorvastatin | 2.89 | [2.89, 3.93] | 0.00% | 0.92 |
| IL-6 |  |  |  |  |
| Total | -2.09 | [-5.84, 1.65] | 99.97% | 0.00 |
| Supplement |  |  |  |  |
| Atorvastatin | -1.15 | [-0.87, 0.58] | 97.66% | 0.00 |
| Atorvastatin+ routine medication | -4.12 | [-11.46, 3.22] | 99.84% | 0.00 |
| Disease |  |  |  |  |
| Infectious disease | -3.83 | [-10.94, 3.28] | 99.93% | 0.00 |
| Asthma | -0.03 | [-0.13, 0.07] | 70.42% | 0.07 |
| COPD | -0.50 | [-6.78, 5.78] | . | . |
| Duration |  |  |  |  |
| 10≥ days | -5.95 | [-15.94, 4.05] | 99.93% | 0.00 |
| 10<days | 0.07 | [-0.17, 0.32] | 90.49% | 0.01 |
| TNF-α |  |  |  |  |
| Total | -0.20 | [-0.28, -0.11] | 92.73% | 0.00 |
| Supplement |  |  |  |  |
| Atorvastatin | -0.16 | [-0.24, -0.08] | 83% | 0.02 |
| Atorvastatin+ routine medication | -0.27 | [-0.31, -0.23] | . | . |
| 6MWD |  |  |  |  |
| Total | 0.71 | [0.24, 1.17] | 47.24% | 0.14 |
| Supplement |  |  |  |  |
| Atorvastatin | 1.31 | [0.67, 1.95] | . | . |
| Atorvastatin+ routine medication | 0.47 | [0.08, 0.85] | 0.00 | 0.78 |
| Duration |  |  |  |  |
| 30≥ days | 0.50 | [-0.08, 1.08] | . | . |
| 30< days | 0.80 | [0.16, 1.43] | 57.63% | 0.09 |
| Disease |  |  |  |  |
| COPD | 0.47 | [0.08, 0.85] | 0.00% | 0.78 |
| Pulmonary hypertension | 1.31 | [0.67, 1.95] | . | . |
| CRP |  |  |  |  |
| Total | -4.08 | [ -11.13, 2.97] | 99.99% | 0.00 |
| Disease |  |  |  |  |
| Infectious | -10.14 | [ -27.58, 7.30] | 99.95% | 0.00 |
| Asthma | -0.29 | [ -0.56, -0.02] | 74.13% | 0.05 |
| COPD | -2.28 | [ -7.10, 2.53] | 93.79% | 0.00 |
| Obstructive Sleep Apnea | 2.5 | [ -3.66, 8.66] | .% | . |
| Dose |  |  |  |  |
| 20 mg | -1.71 | [ -1.85, -1.57] | .% | . |
| 40 mg | -4.41 | [ -12.30, 3.48] | 99.99% | 0.00 |
| Duration |  |  |  |  |
| 30≥ days | -13.83 | [ -36.58, 8.91] | 99.88% | 0.00 |
| 30< days | -0.98 | [ -2.97, 1.02] | 99.91% | 0.00 |
| Supplement |  |  |  |  |
| Atorvastatin | -0.19 | [ -0.52, 0.15] | 72.93% | 0.00 |
| Atorvastatin+ routine medication | -7.44 | [ -19.02, 4.15] | 99.99% | 0.00 |
| TC |  |  |  |  |
| Total | -15.24 | [ -28.28, -2.20] | 99.99% | 0.00 |
| Disease |  |  |  |  |
| Bronchitis | -15.00 | [ -32.26, 2.26] | .% | . |
| Asthma | -19.27 | [ -39.69, 1.14] | 100% | 0.00 |
| COPD | -14.60 | [ -42.69, 13.49] | .% | . |
| Obstructive Sleep Apnea | -0.89 | [ -1.49, -0.29] | .% | . |
| Supplement |  |  |  |  |
| Atorvastatin | -18.29 | [ -34.87, 1.70] | 99.94% | 0.00 |
| Atorvastatin+ routine medication | -1.71 | [ -1.79, -1.63] | 0.00% | 0.37 |
| HDL |  |  |  |  |
| Total | +1.71 | [ -0.63, 4.04] | 99.95% | 0.00 |
| Disease |  |  |  |  |
| Asthma | +2.17 | [ -0.97, 5.31] | 99.98% | 0.00 |
| COPD | +2.25 | [ -3.46, 7.96] | .% | . |
| Obstructive Sleep Apnea | -0.31 | [ -0.77, 0.15] | .% | . |
| Supplement |  |  |  |  |
| Atorvastatin | +2.15 | [ -1.03, 5.32] | 99.38% | 0.00 |
| Atorvastatin+ routine medication | -0.19 | [ -0.22, -0.16] | 0.00% | 0.40 |
| LDL |  |  |  |  |
| Total | -21.48 | [ -30.82, -12.14] | 99.12% | 0.00 |
| Disease |  |  |  |  |
| Bronchitis | -21.00 | [ -24.57, -17.43] | .% | . |
| Asthma | -24.25 | [ -42.82, -5.68] | 97.29% | 0.00 |
| COPD | -28.98 | [ -57.21, -0.76] | 93.15% | 0.00 |
| pulmonary hypertension | -15.14 | [ -31.63, 1.36] | 99.67% | 0.00 |
| Dose |  |  |  |  |
| 20 mg | -28.92 | [ -55.83, -2.02] | 98.02% | 0.00 |
| 40 mg | -19.04 | [ -28.99, -9.09] | 98.10% | 0.00 |
| Supplement |  |  |  |  |
| Atorvastatin | -19.07 | [ -28.63, -9.52] | 99.19% | 0.00 |
| Atorvastatin+ routine medication | -28.98 | [ -57.21, -0.76] | 93.15% | 0.00 |
| TG |  |  |  |  |
| Total | -9.28 | [ -20.76, 2.20] | 100% | 0.00 |
| Disease |  |  |  |  |
| Asthma | -13.03 | [ -28.99, 2.93] | 100% | 0.00 |
| COPD | -2.65 | [ -16.11, 10.81] | .% | . |
| Obstructive Sleep Apnea | +0.10 | [ -0.25, 0.45] | .% | . |
| Supplement |  |  |  |  |
| Atorvastatin | -12.93 | [ -28.99, 3.12] | 99.98% | 0.00 |
| Atorvastatin+ routine medication | -0.27 | [ -0.31, -0.23] | 0.00% | 0.73 |

**Supplementary Table 2.** Atorvastatin Effects on Clinical Parameters with 95% CIs by Subgroup Analysis

| FEV1 |  |  |  |  |
| --- | --- | --- | --- | --- |
| Total | -0.01 | [-0.51, 0.48] | 87.95% | 0.00 |
| Disease |  |  |  |  |
| Bronchial Hyperresponsiveness | -0.31 | [-1.13, 0.52] | .% | . |
| Asthma | -0.02 | [-0.86, 0.82] | 9.83% | 0.00 |
| COPD | 0.34 | [-0.30, 0.98] | 74.72% | 0.01 |
| pulmonary hypertension | -1.22 | [-1.85, -0.59] | .% | . |
| Supplement |  |  |  |  |
| Atorvastatin | 0.04 | [-0.51, 0.58] | 83.93% | 0.00 |
| Atorvastatin+ routine medication | -0.05 | [-1.02, 0.92] | 91.49% | 0.00 |
| Duration |  |  |  |  |
| 30≥ days | 0.38 | [-0.25, 1.02] | 69.46% | 0.05 |
| 30< days | -0.15 | [-0.78, 0.48] | 89.67% | 0.00 |
| Dose |  |  |  |  |
| 20 mg | -0.28 | [-0.70, 0.15] | 0.00% | 0.93 |
| 40 mg | 0.04 | [-0.56, 0.65] | 90.42% | 0.00 |
| RV/TLC |  |  |  |  |
| Total | -2.86 | [-6.53, 0.82] | 51.95% | 0.15 |
| Disease |  |  |  |  |
| Asthma | -2.78 | [-7.09, 1.53] | 73.03% | 0.05 |
| COPD | -3.70 | [-15.35, 7.95] | .% | . |
| Supplement |  |  |  |  |
| Atorvastatin | -2.78 | [-7.09, 1.53] | 73.03% | 0.05 |
| Atorvastatin+ routine medication | -3.70 | [-15.35, 7.95] | .% | . |
| Duration |  |  |  |  |
| 30≥ days | -0.60 | [-3.72, 2.52] | .% | . |
| 30< days | -4.91 | [-8.01, -1.81] | 0.00% | 0.83 |
| FEV1/FVC |  |  |  |  |
| Total | -0.27 | [-4.10, 3.55] | 87.73% | 0.00 |
| Disease |  |  |  |  |
| Asthma | 1.54 | [-0.70, 3.79] | 58.68% | 0.08 |
| Pulmonary hypertension | -4.10 | [-6.97, -1.23] | .% | . |
| Supplement |  |  |  |  |
| Atorvastatin | -0.12 | [-3.95, 3.71] | 91.41% | 0.00 |
| Atorvastatin+ routine medication | -26.20 | [-75.61, 23.21] | .% | . |
| Duration |  |  |  |  |
| 30≥ days | 0.50 | [-0.99, 1.99] | .% | . |
| 30< days | -1.01 | [-7.73, 5.71] | 88.46% | 0.00 |
| FVC |  |  |  |  |
| Total | 1.63 | [-1.15, 4.40] | 94.06% | 0.00 |
| Disease |  |  |  |  |
| Asthma | 0.60 | [-1.18, 2.38] | 84.32% | 0.00 |
| Pulmonary hypertension | 6.40 | [3.55, 9.25] | .% | . |
| Supplement |  |  |  |  |
| Atorvastatin | 2.47 | [-0.63, 5.57] | 85.35% | 0.00 |
| Atorvastatin+ routine medication | -0.67 | [-0.81, -0.53] | .% | . |
| Duration |  |  |  |  |
| 30≥ days | 0.90 | [-0.55, 2.35] | .% | . |
| 30< days | 1.74 | [-2.21, 5.69] | 95.10% | 0.00 |
| FEF25-75 |  |  |  |  |
| Total | 4.99 | [-1.15, 11.14] | 88.24% | 0.00 |
| Disease |  |  |  |  |
| Asthma | -0.17 | [-0.29, -0.05] | .% | . |
| COPD | 8.35 | [4.75, 11.95] | 0.00% | 0.40 |
| Duration |  |  |  |  |
| 30≥ days | 6.69 | [1.42, 11.96] | .% | . |
| 30< days | 4.50 | [-5.25, 14.25] | 93.65% | 0.00 |
| Morning PEF |  |  |  |  |
| Total | -5.55 | [ -11.62, 0.53] | 0.00% | 0.69 |
| Duration |  |  |  |  |
| 30≥ days | -2.86 | [ -11.30, 5.58] | 0.00% | 0.47 |
| 30< days | -8.44 | [ -17.19, 0.31] | 0.00% | 0.72 |
| Supplement |  |  |  |  |
| Atorvastatin | -6.90 | [ -19.05, 5.25] | .% | . |
| Atorvastatin+ routine medication | -5.10 | [ -12.11, 1.92] | 0.00% | 0.50 |
| Evening PEF |  |  |  |  |
| Total | -8.72 | [ -14.96, -2.47] | 0.00% | 1.00 |
| Duration |  |  |  |  |
| 30≥ days | -8.24 | [ -16.82, 0.34] | 0.00% | 0.95 |
| 30< days | -9.25 | [ -18.36, -0.15] | 0.00% | 0.86 |
| Supplement |  |  |  |  |
| Atorvastatin | -8.50 | [ -21.03, 4.03] | .% | . |
| Atorvastatin+ routine medication | -8.79 | [ -15.99, -1.59] | 0.00% | 0.97 |
| O2 saturation |  |  |  |  |
| Total | 0.45 | [ -0.05, 0.95] | 53.81% | 0.07 |
| Disease |  |  |  |  |
| COVID | 1.95 | [ 0.30, 3.60] | .% | . |
| COPD | +1.91 | [ -1.30, 5.12] | 98.96% | 0.00 |
| Duration |  |  |  |  |
| 30≥ days | +0.51 | [ 0.10, 0.92] | .% | . |
| 30< days | +2.40 | [-0.62,5.43] | 94.41% | 0.00 |

**Supplementary Figure S1.** Sensitivity Analyses Excluding Smaller Studies: Impact on Effect Estimates and Uncertainty Highlighting Potential Bias in Selected Parameters.


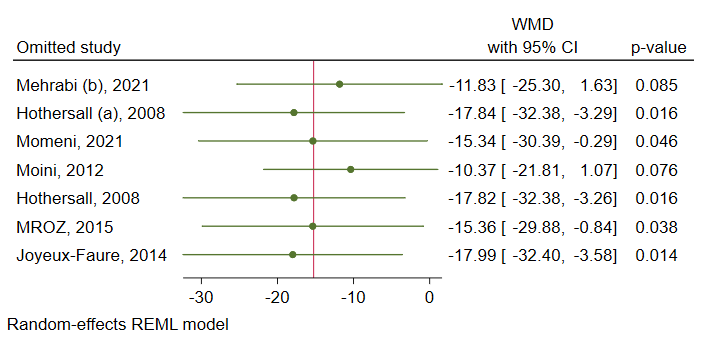

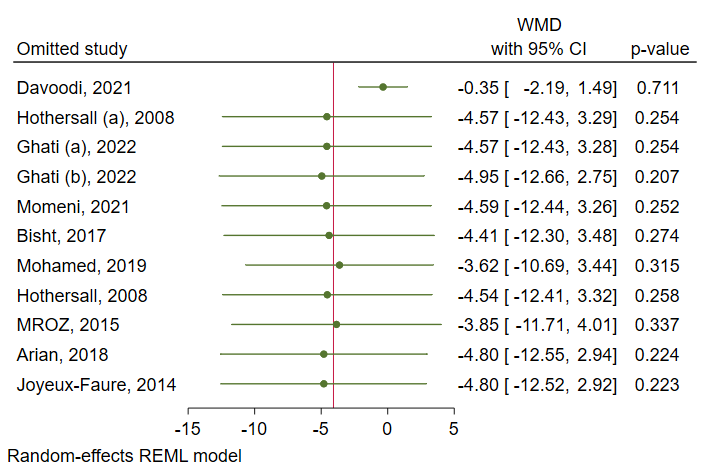

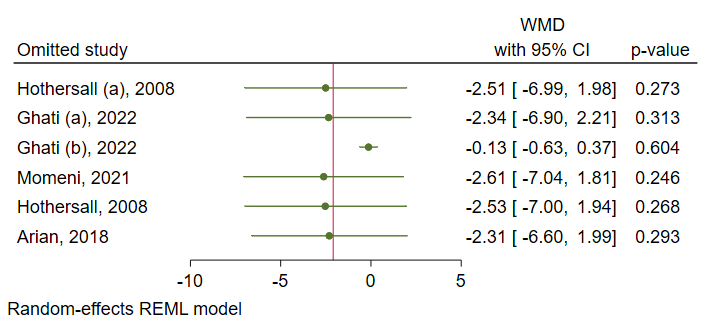


IL-6

TNF-α

**
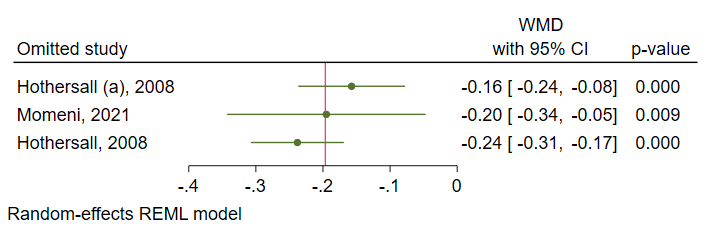
**

CRP

TC

HDL

TG


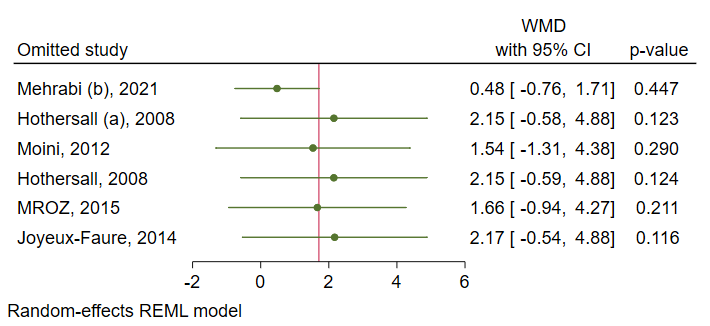

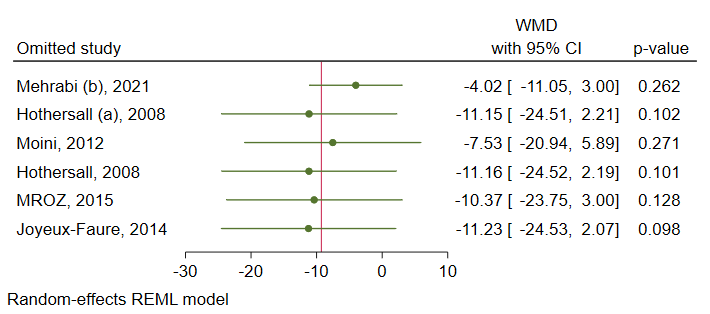


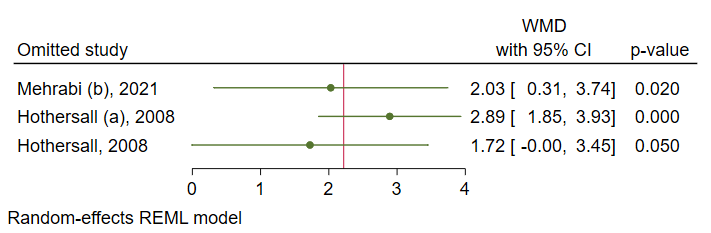

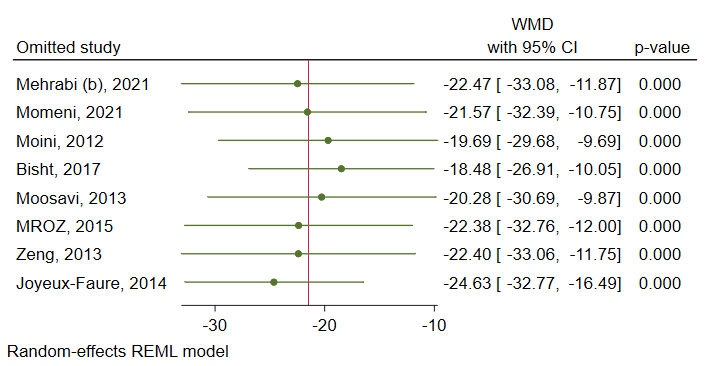


AST

LDL

ALT


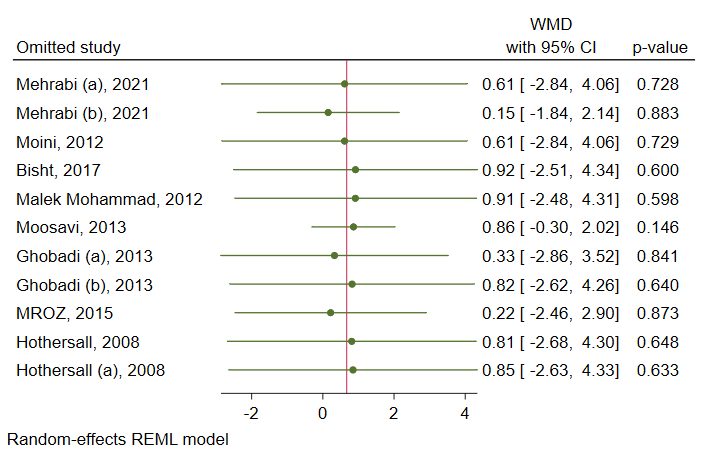

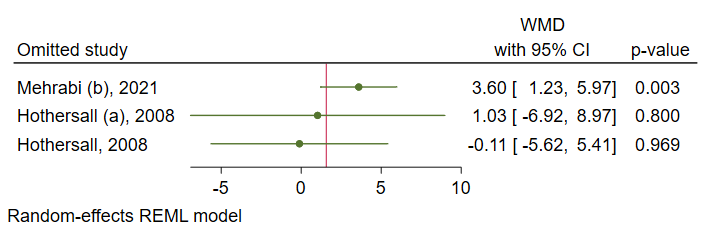


FEV1


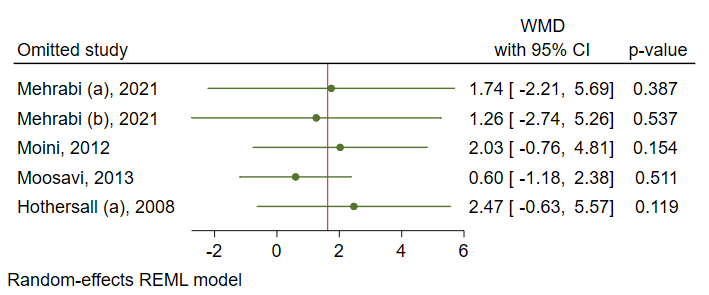

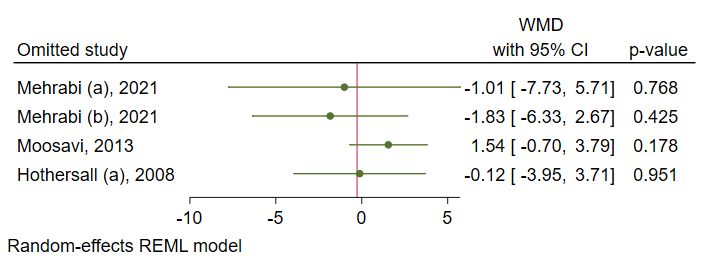


FEV1/FVC

FVC


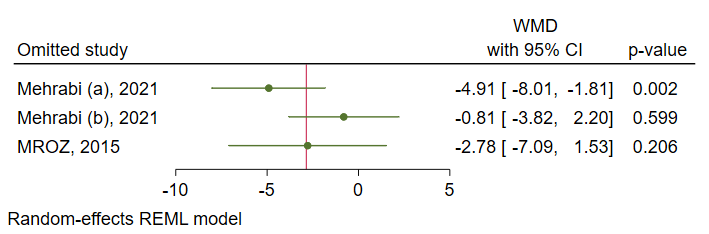

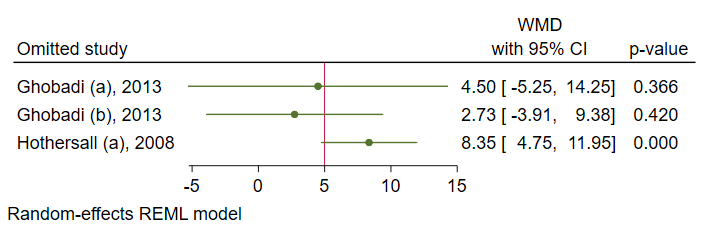


RV/TLC75

FEF2575


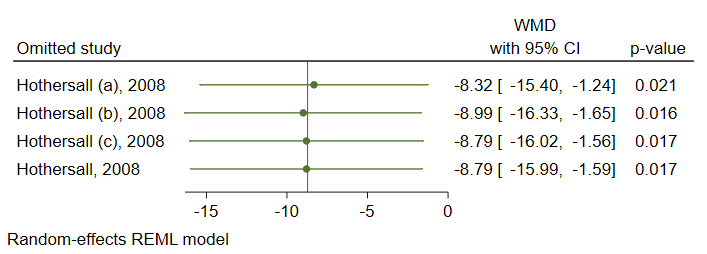

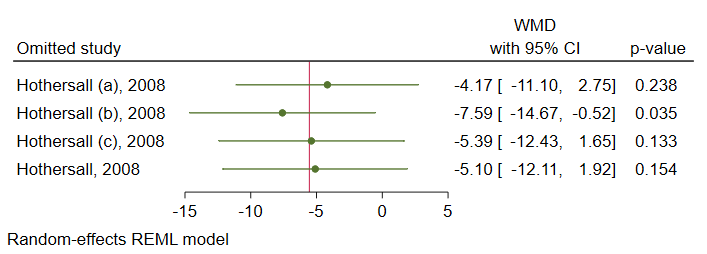


morningPEF

eveningPEF


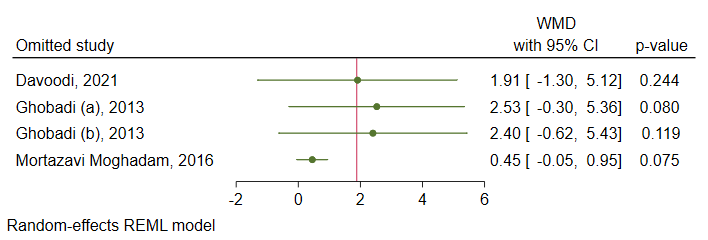


O2Sat
